# Eye Movements Actively Reinstate Spatiotemporal Mnemonic Content

**DOI:** 10.3390/vision3020021

**Published:** 2019-05-18

**Authors:** Jordana S. Wynn, Kelly Shen, Jennifer D. Ryan

**Affiliations:** 1 Rotman Research Institute, Baycrest, 3560 Bathurst St., Toronto, ON M6A 2E1, Canada; 2 Department of Psychology, University of Toronto, 100 St George St., Toronto, ON M5S 3G3, Canada; 3 Department of Psychiatry, University of Toronto, 250 College St., Toronto, ON M5T 1R8, Canada

**Keywords:** eye tracking, eye movements, gaze, memory, retrieval, vision, aging

## Abstract

Eye movements support memory encoding by binding distinct elements of the visual world into coherent representations. However, the role of eye movements in memory retrieval is less clear. We propose that eye movements play a functional role in retrieval by reinstating the encoding context. By overtly shifting attention in a manner that broadly recapitulates the spatial locations and temporal order of encoded content, eye movements facilitate access to, and reactivation of, associated details. Such mnemonic gaze reinstatement may be obligatorily recruited when task demands exceed cognitive resources, as is often observed in older adults. We review research linking gaze reinstatement to retrieval, describe the neural integration between the oculomotor and memory systems, and discuss implications for models of oculomotor control, memory, and aging.

## 1. Eye Movements and Memory Encoding

The visual world is stunningly complex and taking it all in is no easy feat. Since our retina limits visual details mostly to the high-acuity fovea, we must move our eyes continuously to encode the world around us. Several times a second, visual items compete for our attention on the basis of exogenous and endogenous signals, with the winner determining which item will be selected for fixation and further processing. Models of overt visual attention (i.e., eye movements) capitalize on these features, using them to predict how real human observers will explore a given visual scene. Some notable selective attention models, such as the saliency map model [1], predict eye movements based solely on low-level (i.e., salient) visual features such as intensity, color, and edge orientation, and do so significantly better than chance. However, the power of purely bottom-up saliency-based models to predict naturalistic viewing is limited, with other work suggesting that endogenous features such as task instructions, e.g., “estimate the ages of the people in the painting” [2] (see also [3]), prior knowledge, e.g., an octopus does not belong in a barnyard scene [4], and viewing biases, e.g., the tendency to view faces and text [5] (see also [6]) can also be used to predict gaze allocation and to improve the performance of saliency-based models [6,7,8,9]. The combined influence of these cognitive factors on viewing can be summed into “meaning maps”, an analogue to saliency maps generated by crowd sourcing ratings of “meaningfulness” (informativeness + recognizability) for each region of a scene [10]. When compared directly, meaning maps significantly outperform saliency maps in predicting eye movements during naturalistic scene viewing, suggesting that visual saliency alone is insufficient to model human gaze behavior.

**Figure 1:**
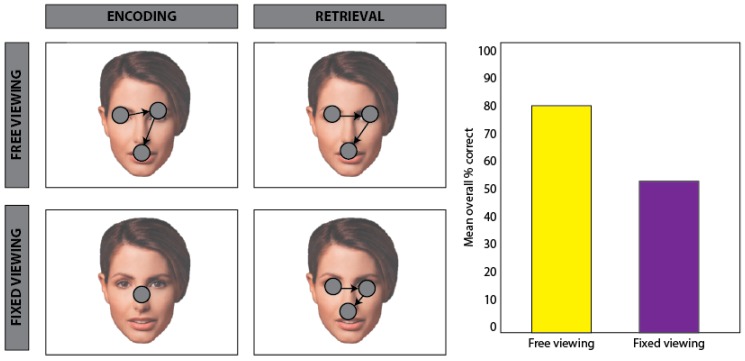
Schematic of encoding-related eye movement effects. Adapted from Henderson, William, and Falk, 2005 [12]. Participants viewed, and were subsequently tested on their memory for, a series of faces. During encoding (**left** column), participants were presented with images of faces. In the free viewing conditions (row 1, **left**), participants were able to move their eyes freely during learning, whereas in the fixed viewing condition (row 2, **left**), participants were required to maintain central fixation. During a recognition test (**right**), participants were presented with repeated and novel faces under free viewing conditions and were required to make an old/new recognition response. The mean percentage of correctly identified faces was significantly lower for faces encoded under the fixed viewing condition compared to faces encoded under the free viewing condition, suggesting that eye movements facilitate the binding of stimulus features at encoding for subsequent memory.

Combined evidence from exogenous and endogenous viewing models suggests that real-world viewing behavior integrates both bottom-up and top-down signals to support selective attention. The results of this selection process have critical implications, not only for attention and perception, but also for memory. Since we cannot encode the entirety of the visual environment at once, fixations and saccades facilitate the alternating encoding and selection of relevant stimulus features. Where we look thus largely determines what we encode into memory and, as a result, what information is available for retrieval. Accordingly, behavioral findings show that recognition accuracy is significantly greater for images encoded under free viewing conditions compared to restricted viewing conditions [11,12]; see Figure 1. Furthermore, for images encoded under free viewing conditions, recognition accuracy is significantly correlated with the amount of visual sampling, i.e., mean number of fixations [13]. However, in cases of amnesia, in which a severe and lasting memory deficit arises due to damage to the hippocampus and its extended system (including the fornix, mammillary bodies and anterior thalami), the relationship between the amount of visual sampling and subsequent recognition is absent [13,14]. These case studies suggest that encoding-related eye movements support the accumulation and integration of visual information into a cohesive memory representation. Expanding on this work, recent neuroimaging findings with healthy young adults indicate that the amount of visual exploration during encoding predicts neural activity in the hippocampus, further suggesting that encoding-related eye movements are involved in the development of lasting memories [15]. Moreover, in nonhuman primates, saccades have been shown to modulate hippocampal neuron spiking and theta phase activity during visual exploration, and this modulation has been linked to memory formation [16,17]. In humans, evidence from intracranial recordings indicates that temporal coordination between saccades and alpha oscillatory activity in brain regions supporting scene perception and memory predicts successful memory encoding [18]. Thus, taken together, findings from behavioral, neuropsychological, neuroimaging, and electrophysiological research converge on a key role for eye movements in selecting and integrating visual information in the service of memory encoding. However, research has yet to reach consensus on whether and how eye movements support memory retrieval.

## 2. Eye Movements and Memory Retrieval

Although the role of eye movements in memory encoding has been well established, a relatively smaller literature suggests that eye movements may also reflect, and be guided by, the contents of memory at retrieval. For example, several studies have shown that humans make fewer fixations and view fewer regions during the examination of repeated images compared to novel images [19,20], and other studies have shown that eye movements during retrieval are disproportionately drawn to regions of a stimulus that reflect a previously learned association [21] or have changed from a prior viewing [20]. For example, a study by Hannula and Ranganath (2009) [21] found that following a scene cue, participants disproportionately directed viewing to a face that had been associated with that scene in a prior viewing compared to other previously viewed, but unpaired, faces. Moreover, this viewing effect was correlated with increased activity in the hippocampus, suggesting that eye movements reflect hippocampally-mediated memory regarding the relations among items. Notably, this eye movement-based expression of memory for relations, and associated activity in the hippocampus, was observed regardless of whether viewers had explicit recognition of the appropriate scene-face pairing. Thus, eye movements may reveal the contents of memory in an obligatory manner, such that it can occur outside of conscious awareness.

Whereas several studies have provided evidence that eye movements can reflect the contents of memory, other studies suggest that memory retrieval can *directly guide* further viewing behavior. For example, Bridge, Cohen and Voss (2017) [22] found that following the retrieval of one item in an array, participants strategically directed viewing to the other items in the display, presumably to allow for re-examination of previously studied items that were not as strongly represented in memory. This effect was associated with functional connectivity between the hippocampus and fronto-parietal control regions, supporting a possible mechanism by which memory retrieval might guide viewing behavior. Indeed, network analyses and computational modeling of anatomical connections between the hippocampus and oculomotor control regions (including frontal eye fields) indicate that there are several pathways by which memories might guide ongoing visual exploration [23,24]. However, whether eye movements can in turn influence memory retrieval remains unclear.

The characterization of eye movements as a *passive* reflection of mnemonic content has been generally agreed upon, and their guidance by memory in particular, though less extensively studied, has likewise received some support. However, the notion that eye movements *actively* facilitate memory retrieval has been a subject of contentious debate. In the sections that follow, we propose that eye movements play a functional role in retrieval by reinstating the spatial and temporal details of the encoding context, in alignment with past models of endogenous viewing guidance, current theories regarding memory function, and new evidence of memory–oculomotor network interactions.

### Scanpath Theory

The notion that eye movements play a functional role in memory retrieval can be traced back to Hebb’s musings on mental imagery: “If the image is a reinstatement of the perceptual process it should include the eye movements”. Hebb went on to suggest that the oculomotor system, upon being presented with a “part-image”, might serve as a link between perception and imagery by activating the next “part-image” [25,26], an idea that was subsequently formalized by Noton and Stark’s (1971) [27,28] seminal scanpath theory. Based on the observation that participants repeatedly examining a simple line drawing produced similar patterns of eye movements with each viewing, scanpath theory proposed that image features are represented in memory along with the accompanying series of fixations and alternating saccades in a sensory-motor memory trace or *scanpath*. According to scanpath theory, recapitulation of the encoding scanpath during repeated viewing (or by extension, imagery) facilitates memory retrieval by comparing presented input with stored memory traces. Though subsequent research would depart from its strict predictions, scanpath theory’s legacy lies in its groundbreaking proposal that eye movements play a functional role in retrieval, an idea that has been critical in shaping current models of memory and visual attention.

**Figure 2:**
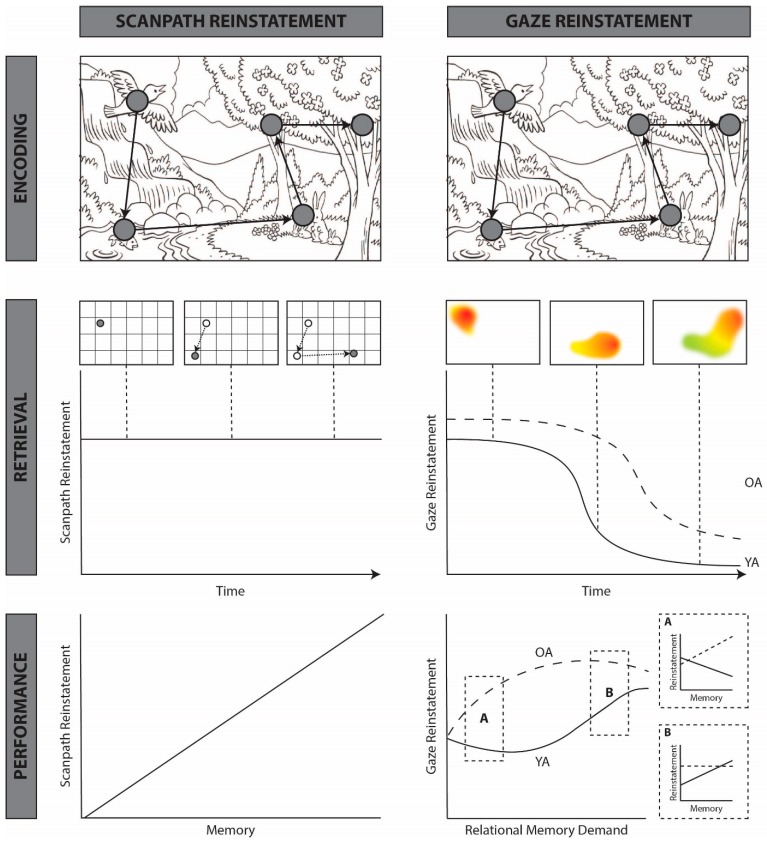
Schematic comparing the predictions of the standard scanpath model (**left**) and the proposed gaze reinstatement model (**right**). Scanpath model (**left**): Row 1: a simplified scanpath enacted during the encoding of a line drawing of a scene. The same encoding scanpath is used to illustrate the predictions of both the scanpath model and the gaze reinstatement model (row 2, **right**). Row 2: the predictions of the standard scanpath model regarding retrieval-related viewing. In the present example, retrieval consists of visualization while “looking at nothing”. However, these predictions could similarly apply to repeated viewing of the stimulus. Early tests of scanpath theory used string similarity analyses to measure the similarity between encoding and retrieval fixation sequences [29,30,32]. These methods label fixations based on their location within predefined interest areas (often based on a grid, as shown here) and compute the number of transitions required to convert one scanpath into the other. Scanpath theory does not make any predictions regarding scanpath reinstatement over time or with memory decline [27,28]. Row 3: the predictions of the standard scanpath model regarding the relationship between reinstatement and mnemonic performance. The scanpath model predicts that scanpath reinstatement will be positively correlated with mnemonic performance [27,28]. Gaze reinstatement model (**right**): Row 1: a simplified scanpath enacted during the encoding of a line drawing of a scene. This is the same scanpath that is used to make predictions regarding the scanpath model (top **left**). Row 2: the gaze reinstatement model proposes that retrieval-related viewing patterns broadly reinstate the temporal order and spatial locations of encoding-related fixations. In the present example, gaze reinstatement decreases across time. This would be expected in the case of image recognition, wherein reinstatement declines when sufficient visual information has been gathered, e.g., [27,28,35,47], or in the case of image visualization, when the most salient parts of the image have been reinstated, e.g., [43,48]. The duration of gaze reinstatement would be expected to change based on the nature of the retrieval task (e.g., visual search, [37]). The gaze reinstatement model additionally predicts that reinstatement will be greater and extended in time for older adults (OA), relative to younger adults (YA) [36,37]. Row 3: The gaze reinstatement model (**right**) predicts that the relationship between reinstatement and mnemonic performance is modulated by memory demands (i.e., memory for spatial, temporal, or object-object relations) and memory integrity (indexed here by age). When relational memory demands are low (A), older adults, and some low performing younger adults, use gaze reinstatement to support mnemonic performance [36]. As demands on relational memory increase (B), the relationship between reinstatement and mnemonic performance in older adults plateaus, whereas younger adults use gaze reinstatement to support performance [36,43]. Based on findings from the compensation literature [49], we predict that as relational memory demands overwhelm older adults, gaze reinstatement will not be sufficient to support performance and will thus decline, whereas in younger adults, the relationship between gaze reinstatement and mnemonic performance would plateau before eventually declining as well.

Although interpretations vary widely, scanpath theory makes two key predictions: (1) remembered stimuli should be accompanied by repetition of the scanpath enacted during encoding (see Figure 2, middle left), and (2) similarity between scanpaths during encoding and retrieval should predict memory performance (see Figure 2, bottom left). Confirming the first prediction, early studies using eye movement monitoring demonstrated that scanpaths are more similar for the same subject viewing the same image than for the same subject viewing different images or different participants viewing the same image [29,30,31,32]. Using string similarity metrics (see Figure 2 caption for description), these studies showed that eye movements during repeated stimulus presentations reinstate both the spatial locations and temporal order of eye movements enacted during novel viewing of those same stimuli. Moreover, repetition of the encoding scanpath during repeated viewing is greater than would be expected based on chance or visual saliency [33,34,35], or based on subject-invariant viewing biases, such as the tendency to fixate the center of an image [36,37], suggesting that eye movements reflect the content of memory above and beyond image- or subject-specific idiosyncrasies.

According to scanpath theory, retrieval-related eye movements not only reflect memory, but also play a critical role in supporting it. Consistent with this hypothesis, recent work has demonstrated that similarity between encoding and test fixations is greater for recognition hits than misses [38,39] and for “remember” responses (associated with a stronger recollection-based memory) relative to “know” responses (associated with a weaker, familiarity-based memory) [35]. These findings suggest that eye movement reinstatement is related to both objective memory and the subjective sense of memory strength. Several studies have shown that across a variety of tasks, the reinstatement of encoding-related eye movements supports memory retrieval [36,37,40,41,42,43], whereas maintaining fixation impairs it [12,42]. Yet, despite supporting evidence, and a revived interest and rapidly growing literature on eye-movement-based memory effects, scanpath theory has largely fallen out of favor in recent years.

According to scanpath theory, image recognition is achieved via “an alternating sequence of sensory and motor memory traces” matching perceived image features to remembered features, a process that is theoretically accompanied by complete reinstatement of the encoding scanpath [27,28]; see Figure 2 middle left. However, few studies have actually examined or found evidence for complete scanpath repetition [33,37,41,44]. In their initial experiments, Noton and Stark (1971) [27] noted that only initial fixations were reinstated during repeated stimulus viewings and only on 65% of trials, on average. Other studies have similarly shown that the temporal sequence of encoding fixations is not recapitulated in full during retrieval. Evidence of the reinstatement of previously sampled spatial regions is similarly varied, with some studies defining spatial similarity based on screen quadrants, e.g., [42,45,46,47] and others using more strictly defined grid patterns, e.g., [32,41] or experimenter-defined areas of interest, e.g., [30,45]. Despite wide variance in definitions and measures of scanpath similarity, multiple studies have found evidence for some amount of eye movement-based reinstatement during repeated stimulus presentations and retrieval. Yet, several critical questions remain unanswered. Primarily, how is the reinstatement of encoding-related gaze patterns related to underlying mnemonic content, and under what conditions does such reinstatement support memory retrieval?

## 3. A New Theory of Functional Gaze Reinstatement

We propose that eye movements support online memory retrieval; that is, active retrieval that continuously updates as viewers move their eyes, by broadly reinstating the spatiotemporal encoding context based on the demands of the task and availability of cognitive resources; see Figure 2, right panel. Such reinstatement may include the spatial locations of salient encoded stimuli and/or the temporal order in which they were encoded and supports memory retrieval by reactivating additional features (e.g., semantic, perceptual) associated with sampled locations.

Whereas others have suggested that the reinstatement of any feature of the encoding event (including conceptual, linguistic, and visual features) is sufficient to reinstate the other features of that event (see [50]), we propose that gaze reinstatement specifically supports the reinstatement of the spatiotemporal context, which in turn supports the retrieval of associated event features. Indeed, spatial and temporal information have been proposed to play a foundational role in organizing and structuring percepts and memories [51,52], and the reinstatement of the encoding context has been proposed to facilitate the reinstatement of associated stimulus/event details [53,54]. Accordingly, we further propose that gaze reinstatement and the relationship between gaze reinstatement and memory retrieval are flexibly modulated by both the mnemonic demands of the task and the integrity of memory functions, such that gaze reinstatement is recruited to support memory when the mnemonic demands of the task exceed memory capacity.

Although eye movement reinstatement has traditionally been conceived as a fixation-by-fixation reactivation of associated stimulus features, widespread evidence of eye movement reinstatement across a variety of tasks and using different similarity measures suggests that the underlying mechanism is insensitive to small deviations in spatial or temporal scale. In other words, the mnemonic benefits conferred by reinstatement do not appear to rely on the reinstatement of the precise spatial locations or temporal order of encoding-related fixations, or even on complete recapitulation of the encoding scanpath. Therefore, we advocate for use of the term “gaze reinstatement” to broadly describe evidence of similarity (regardless of the measure employed) that is greater than would be expected by chance or based on subject- or image-specific characteristics. Regardless of the similarity measure used, gaze reinstatement, as defined here, reflects a specialized role for the reinstatement of subject- and image-specific gaze patterns in the retrieval of spatiotemporal contextual information. These gaze patterns need not be complete or exact reinstatements of the motor program enacted at encoding, but must contain some overlapping spatial and temporal information in order to facilitate memory retrieval (see Figure 2, middle right). In the following sections, we provide support for our proposal, drawing on evidence from eye movement monitoring, neuropsychology, neuroimaging, and emerging computational modeling to elucidate the relationship between eye movements, memory retrieval, and the neural systems involved in storing spatiotemporal relations.

## 4. How Does Gaze Reinstatement Support Memory Retrieval?

### 4.1. Spatial Reinstatement

**Figure 3:**
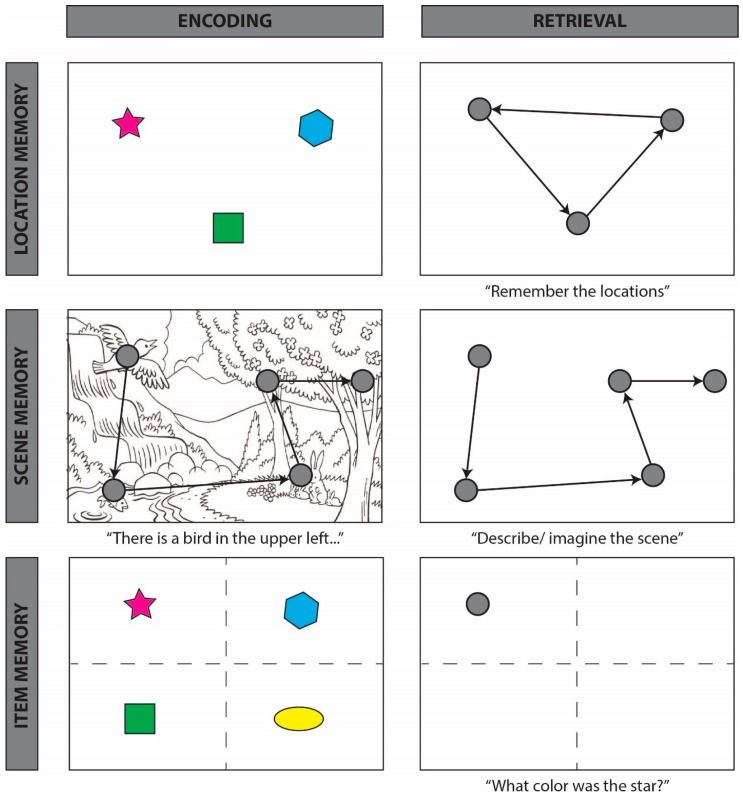
Schematic of “looking at nothing” behavior, whereby participants reinstate encoding-related eye movements during retrieval in the absence of visual input, across three task types. Row 1 depicts tasks in which participants are required to remember the relative locations of presented objects (**left**). During maintenance (whereby a representation is held in an active state in memory) or retrieval (**right**), participants’ eye movements reinstate the locations and spatial relations among encoded objects, e.g., [36,43]. Row 2 depicts tasks in which participants are required to remember a complex scene that was presented either visually or auditorily (**left**). During retrieval (**right**), participants’ eye movements return to regions that were inspected during encoding, e.g., [48,56]. Row 3 depicts tasks in which participants are required to answer questions or make judgments about previously presented items. During retrieval (**right**), participants look in the region of the scene that previously contained the target item, even when successful task performance does not require the retrieval of the previously observed spatial locations [42,45]. For within-item effects, see [40]; for words, see [46]; such effects persist even after a week-long delay [61].

By nature, scanpaths contain spatial and temporal information regarding the location and order of fixations. But the extent to which prior spatial and temporal information are embedded in the scanpath, and contribute to memory retrieval, remains unclear. Following the tradition of using eye movements to examine the contents of memory, e.g., [55], Ryan and colleagues (2000) [20] showed that removing or moving an item in a previously studied scene resulted in increased viewing to the now empty region that had previously contained that item, despite a lack of salient visual information at that location. Critically, this study provided one of the first demonstrations that viewers use their eyes to reinstate the memory for the exact position of a previously studied object by disproportionately viewing the region previously occupied by that object. Building on this research, several studies have since adopted a “looking at nothing” paradigm; for review, see [50], in which viewing patterns during perception are compared to viewing patterns during a period in which no stimulus is present; see Figure 3. In the absence of visual input, participants, often spontaneously, look at regions of a scene corresponding to locations described auditorily [42,46,56,57,58,59,60], or visually [32,40,41,61]. When cued to recall a previously presented stimulus on a blank screen, for example, participants spend a disproportionate amount of time looking in the screen quadrant in which the stimulus previously appeared relative to the other quadrants, despite them being equally devoid of visual information [41,42,46,56,57,61]. Moreover, preferential viewing of screen regions previously occupied by salient information has been correlated with an array of task performance measures, including imagery vividness [41], reaction time [37,42], memory accuracy [40,60], and change detection performance [36,43], further suggesting that the eye movement-based reinstatement of spatial contextual information supports mnemonic performance.

### 4.2. Temporal Reinstatement

Like spatial reinstatement, research suggests that retrieval-related eye movements can reinstate the temporal order of the encoded scanpath, and that such reinstatement may be functional for memory retrieval. For example, using an old/new recognition task with yoked encoding (where the image is presented one part at a time) and free retrieval, Foulsham and Kingstone (2013) [48] found that participants were more likely to look at previously encoded image regions than non-encoded regions during retrieval, and that this effect followed a temporal trend, such that the region that was presented first at encoding was the region most likely to be viewed at retrieval. Given that image regions high in visual [62,63,64] or semantic [65] saliency are likely to be visited first during encoding, it is perhaps not surprising that these regions are also likely to be visited first during retrieval, as they facilitate the matching of present input with stored memory representations (see Figure 2, bottom right). Indeed, the preservation of temporal order in initial fixations has been widely reported in image recognition tasks [39,41,46,47,64]. Critically, however, the reinstatement of spatial locations and temporal order are often confounded. In order to tease these mechanisms apart, Rondina and colleagues (2017) devised an experiment in which studied items were manipulated in either their spatial locations or temporal order at test [52]. During the study phase, participants were presented with three unique items, presented one at a time in different locations on the screen. During the test phase, items could appear in the same locations, but a different order, or in the same temporal order, but different locations. Whereas changes in the spatial locations of test items did not affect memory for their temporal order, changes in the temporal order of test items did affect memory for their spatial locations. Moreover, changes in the temporal order of test items, but not changes in their spatial locations, lead to increased looking back (to regions previously occupied by presented test items) behavior. Extending previous work, these findings suggest that memory for temporal relations, preserved in eye movements, plays a critical organizing role in memory independent of memory for spatial relations.

Although the reinstatement of initial fixations is consistent with the time-course and cognitive demands of image recognition tasks, gaze reinstatement in more complex tasks such as visual search follows a different trajectory. Notably, targets in repeated search arrays are detected faster [66] and in fewer fixations [67] than targets in novel arrays, thus negating the possibility of complete scanpath repetition. Moreover, unlike recognition memory, visual search relies on the dual processes of image recognition and target detection, necessarily in that sequence. Accordingly, we hypothesized that repeating search arrays would result in speeded target detection and incomplete repetition of the encoding scanpath [37,68]. To evaluate the latter prediction, we measured the similarity between contiguous subsets of corresponding (same subject and image) novel and repeated viewing fixations at multiple time points across the search scanpath. In line with our hypothesis, gaze reinstatement during repeated search arrays was limited to the first few and last few search fixations, consistent with their respective roles in image recognition and target detection. Moreover, the reinstatement of initial fixations was positively predictive of repeated search performance whereas the reinstatement of fixations from the middle of the encoding scanpath was negatively predictive of repeated search performance. These findings suggest that recapitulation of the first few fixations in the scanpath provides enough information to facilitate the retrieval of the target location and subsequent target detection, while eliminating unnecessary (middle) fixations from the scanpath (i.e., those that do not contribute to image recognition or target detection). Taken together with previous work, these findings support an earlier suggestion made by Noton and Stark (1971) that the scanpath may prioritize “essential fixations at major points on the path” [27], such that only fixations that facilitate the current task goals are reinstated.

## 5. When Does Gaze Reinstatement Support Memory Retrieval?

Through recent theoretical and methodological advances, it is now clear that the reinstatement of encoding-related gaze patterns during memory maintenance and retrieval supports mnemonic performance by reinstating the spatiotemporal encoding context. When participants spontaneously reinstate encoding-related gaze patterns, memory performance benefits. On the other hand, when participants fail to reinstate encoding-related gaze patterns, either spontaneously or through restrictive gaze manipulations, memory suffers. Although ample evidence suggests that changes in gaze reinstatement can affect changes in memory performance, relatively fewer studies have investigated whether changes in memory can affect changes in gaze reinstatement. This question can be answered in two ways: (1) by looking at reinstatement in tasks in which memory demands are manipulated, for example by increasing memory load or delay, and (2) by looking at reinstatement in cases of memory impairment, such as that seen in healthy aging or disease. Although further research is needed to elucidate the conditions under which gaze reinstatement supports memory performance, current evidence suggests that gaze reinstatement is recruited to support performance when demands on memory exceed cognitive capacity, either by virtue of increased task difficulty or often-observed age-related memory decline.

### 5.1. Effects of Task Demands

In order to investigate how task-related changes affect visual sampling, and task performance, we examined gaze reinstatement across two experiments in which participants studied arrays of abstract objects and subsequently performed a change detection task on the same objects. Critically, the study and test phases were separated by a delay period of variable length, ranging from 650 ms–20 s across the two studies [36,43]. The relationship between gaze reinstatement and change detection in younger adults depended on, and increased with, the length of the delay, such that gaze reinstatement was negatively correlated with performance at short delays and positively correlated with performance at long delays. That is, when mnemonic demands were low (i.e., when objects only had to be held in memory over brief delays), only younger adults who performed poorly on the task recruited gaze reinstatement [36], but when mnemonic demands were high (i.e., when objects had to be held in memory over long delays, [43]), gaze reinstatement supported performance in younger adults by maintaining object locations in memory, (see Figure 2, bottom right). Indeed, varying task demands may explain several discrepancies in findings of gaze reinstatement. For example, shuffling the order in which encoded images are presented at test impairs memory when those images are complex stimuli [45], but has no effect when the images are simple grid patterns [48]. Together, these findings suggest that gaze reinstatement may be necessarily recruited when demands on memory exceed cognitive resources.

### 5.2. Effects of Memory Decline

Given that gaze reinstatement increases in response to increased task demands, we might expect similar effects in populations in whom memory function is already impaired, such as in older adults. Yet, research suggests that many endogenous viewing effects are impervious to age-related changes. For example, younger and older adults similarly benefit from intentional viewing instructions [69], active viewing (e.g., user-controlled) viewing versus passive viewing (e.g., following a moving window) [70], and free (versus constrained) viewing [11]. In line with findings of preserved top-down viewing guidance in older adults, several studies have now provided evidence for age-related increases in gaze reinstatement [36,37,71], even when performance is otherwise unaffected [36,37]. During repeated visual search, for example, older adults reinstate more initial encoding fixations relative to younger adults, although both groups show similar repetition-related increases in search efficiency [37]. Thus, whereas younger adults need only to reinstate a few encoding-related fixations in order to recognize the image and subsequently retrieve the target location from memory, older adults must refixate more of the presented image in order to gather sufficient information for comparison with internal mnemonic representations. Likewise, when older adults were tested on the previously described change detection task, they showed significantly greater delay-period gaze reinstatement than younger adults, despite similar performance [36]. This effect was strongest at short delays and decreased with increasing delay, while the opposite effect was observed for younger adults (i.e., gaze reinstatement was negatively correlated with performance at short delays, but a positive relationship was observed with increasing delay). Consistent with evidence of widespread memory dysfunction in age [72,73], these findings suggest that older adults might recruit gaze reinstatement to support mnemonic performance when task demands exceed cognitive resources. Moreover, drawing on related findings of age-related compensation, whereby older adults over-recruit cognitive and neural resources relative to younger adults to achieve similar levels of performance [49,74,75], these findings reflect part of a larger pattern of task and memory effects on gaze reinstatement. Like compensation, gaze reinstatement may follow an inverted u-shaped curve, increasing with increasing memory demands until a critical point and then decreasing. In other words, when a task is too easy or too difficult relative to memory capacity, gaze reinstatement may be either not necessary or not available to support performance; see Figure 2, bottom right. Finally, although the focus here in on aging, we expect similar, or perhaps more graded effects, in other memory-impaired populations such as those with dementia or amnesia.

### 5.3. What Are the Conditions under Which Gaze Reinstatement Supports Memory Retrieval?

Although the described evidence suggests that younger and older adults alike spontaneously reinstate encoding-related gaze patterns to support memory maintenance and retrieval, several studies have failed to observe these effects. Examined through the lens of the current proposal, these findings help further inform the conditions under which gaze reinstatement occurs and supports memory retrieval. For example, a study by Johansson, Holsanova, Dewhurst and Holmqvist (2012) [59] showed that participants who were forced to maintain fixation during encoding did not similarly maintain fixation at retrieval (oral recall) when they were permitted to freely move their eyes over a blank screen; see also [12], Figure 1. This finding was interpreted as evidence that, contrary to prior research, retrieval-related eye movements are not reinstatements of the specific eye movements enacted during encoding. Critically, eye movements made during retrieval did correspond to the spatial locations described by the participants during oral recall. When considered in light of the present proposal, these findings suggest that retrieval-related fixations may not reinstate encoding-related fixations when those fixations are uninformative, as in the case of fixed viewing, but, rather, reinstate the spatial context of the studied stimulus, which can be encoded overtly or covertly. Indeed, in a second experiment, participants who were forced to maintain fixation during retrieval showed significantly poorer (less detailed) recall than participants who were able to freely view the blank screen, further suggesting that although retrieval-related fixations may not precisely replicate the fixations that occur at encoding, reinstating the spatial context via gaze shifts plays an important role in memory retrieval.

In another study, Damiano and Walther (2019) [39] showed that although encoding-retrieval eye movement similarity was greater for remembered images compared to forgotten images, memory performance could only be predicted from gaze patterns at encoding, but not retrieval. Moreover, recognition hit rates were significantly reduced when viewing was restricted during encoding, whereas restricting viewing at retrieval had no effect. The authors interpreted these findings as evidence that eye movements during test play no role in memory retrieval. Critically, however, test fixations were predictive of memory when they were taken from the first 600 ms of the test phase. This finding is consistent with previous research suggesting that the reinstatement of initial fixations supports image recognition [27,28,35,37,48], and is in line with our proposal that the relationship between gaze reinstatement and memory may change across time depending on the demands of the task. Finally, although fixed viewing at test did not significantly affect the hit rate, it did significantly increase the rate of false alarms, further suggesting that gaze reinstatement is involved in the comparison of present input with stored memory representations.

Consistent with the present proposal, findings from the described studies suggest that gaze reinstatement, though not necessary for memory retrieval, can support retrieval when task demands exceed cognitive resources by reinstating the spatiotemporal encoding context in line with current goals. Extending this work, other research suggests that by binding and/or comparing spatial and temporal relations among stimulus elements, gaze reinstatement specifically supports performance on tasks that rely on memory for relations. If a task can be accomplished using non-relational mnemonic processes, e.g., semantic memory, [56] and object memory [42,43], gaze reinstatement may not be necessary or useful for performance. For example, using a simple change detection task, Olsen and colleagues (2014) [43] showed that the similarity between eye movements during the study and retention of a set of abstract visual objects correlated significantly with memory for relative, but not absolute object locations. Extending those findings to retrieval, Johansson and Johansson (2013) [42] demonstrated that constraining gaze during a memory test prolonged response times to questions regarding the relative location, but not orientation of studied objects, suggesting that retaining and reinstating the spatial index of encoding is important for recall of inter-object, but not intra-object spatial information. Taken together, these studies suggest that the relations among objects in, and with, the larger spatial context are reinstated via eye movements and may serve as a scaffold for further retrieval of detail-rich memories; for review, see [51].

### 5.4. What Are the Neural Correlates of Gaze Reinstatement?

The notion that spatiotemporal gaze reinstatement supports memory retrieval aligns with the purported role of the hippocampus, a region of the brain that is critical for memory and whose functions are disrupted in amnesia and in aging. It has been suggested that the primary role of the hippocampus is in the obligatory binding of relationships among elements of an encoding event (including spatial and temporal elements), which are stored independently within cortex, in order to form new memories [76,77,78]. Additionally, the hippocampus plays a role in the comparison of incoming relational information to relations already stored in memory in order to form integrated memory traces of complex spatial and temporal episodes [76,79,80,81,82,83,84,85]. Support for the role of the hippocampus in relational memory is well documented in neuropsychological studies, in which amnesic cases show impairments specific to relational memory [20,76,86], electrophysiological studies, in which cells within the hippocampus respond to space [87] and time [88], and neuroimaging studies, in which the hippocampus shows increasing activity in response to increasing task demands [89,90,91,92]. Thus, the hippocampus plays a critical role in the binding and comparison of information across space and time.

Although extensive research has documented the binding and comparison functions of the hippocampus during memory-guided behavior, research using eye movement monitoring suggests that these functions extend to active viewing behavior. As discussed earlier, the hippocampus supports the formation of lasting memories via encoding-related visual exploration [13,15,16,17,18], and the retrieval of those memories by means of memory-guided overt visual attention [22]. Extending these findings to gaze reinstatement, Ryals, Wang, Polnaszek, and Voss (2015) [38] looked at the similarity of eye movements (“exploration overlap”) during study and test for novel and configurally similar computer-generated scenes. Although participants were unable to reliably discriminate between new and similar scenes at test, exploration overlap was significantly greater for similar scenes than novel scenes, and for similar scenes correctly identified as similar than those incorrectly endorsed as new. Notably, trial-by-trial variability in exploration overlap was correlated with activity in the right hippocampus, suggesting that, similar to other eye-movement-based retrieval effects, gaze reinstatement might reflect hippocampal relational memory. Moreover, activity related to exploration overlap was observed in cortical regions including middle frontal gyrus and inferior parietal cortex. Thus, gaze reinstatement may support retrieval by reactivating the spatiotemporal context (via the hippocampus) and, in turn, associated stimulus features (via hippocampal–neocortical interactions), although further research using combined eye tracking and neuroimaging will be required to determine whether this is indeed the case.

In a second study investigating the neural correlates of gaze reinstatement, Bone et al. (2018) [93] had participants study a set of images in preparation for an imagery test in which they were instructed to visualize those same images (on a blank screen) and rate their subjective vividness. Consistent with findings from other “looking at nothing” studies [32,40,41,50,61], gaze patterns during imagery were significantly similar to gaze patterns during encoding, suggesting that even in the absence of visual input, participants reinstate the context of encoding via gaze shifts. Interestingly, gaze reinstatement was significantly correlated with whole-brain neural reinstatement (i.e., similarity between image-specific patterns of brain activity evoked during perception and imagery), which was in turn correlated with subjective vividness ratings and subsequent memory performance. These findings suggest that gaze reinstatement contributes to the construction of a mental image during visualization. Taken together with the findings from Ryals et al. (2015) [38], these results suggest that gaze reinstatement is supported by the same neural mechanisms that support mental imagery and relational memory. However, further research will be required to fully elucidate the neural mechanisms underlying gaze reinstatement across different tasks and populations.

## 6. Implications for Models of Oculomotor Control, Memory, and Aging

Despite much research suggesting that eye movements and memory, and the neural regions underlying them, are intimately related, few models of oculomotor control account for memory. Most popular theories of oculomotor control model the guidance of eye movements based on a “priority map” that combines stimulus-driven features such as luminance, color and contrast with relevant top-down information such as task rules and prior knowledge [1,94,95]. The selection of the location of the next gaze fixation proceeds in a winner-take-all fashion where the peak/largest representation is selected, e.g., [96]. The neural instantiation of the priority map involves neural activity across a network of oculomotor control areas such as the frontal eye fields (FEF), the lateral intraparietal area (LIP), and superior colliculus (SC), which represent the location of an upcoming eye movement [97,98,99,100,101]. Most models also include a form of retention, which discourages the eyes from moving back to recently visited locations. This retention process is often considered as an attentional disengagement, i.e., inhibition of return [94] or mediated by visual working memory [102] via a fronto-parietal network. Yet, even with this retention process, existing models lack the power to fully explain the described effects of gaze reinstatement. These effects include looking at empty regions that previously held a salient stimulus in “looking at nothing” paradigms, and the relationship between gaze reinstatement and subsequent (relational) memory performance, particularly at longer delays. Thus, a comprehensive model of oculomotor control should consider inclusion of regions critical for long-term memory, such as the hippocampus and broader medial temporal lobe.

Recent work has suggested that the hippocampus and larger medial temporal lobe network might modulate activity in regions involved in the computation of visual saliency and selection of saccade targets [103]. Indeed, network analysis of anatomical connections between the hippocampal memory network and oculomotor network in the macaque brain indicates that there are several polysynaptic pathways by which relational memories might guide ongoing visual exploration [23]. Computational modeling of potential functional pathways connecting these two networks further indicates that stimulation of hippocampal nodes identified by the network analysis leads to observable responses in oculomotor control regions including the frontal eye fields [24]. Taken together, these findings point to a potential anatomical pathway by which spatiotemporal relational memories retrieved by the hippocampus may guide gaze reinstatement, and by which gaze reinstatement may support further memory retrieval. Although current evidence supporting the proposed role for functional gaze reinstatement is primarily behavioral, future work using neuroimaging, computational, and analytical techniques may help us to further determine whether feedback from reinstated gaze patterns can act back on the hippocampus and other memory regions to support and strengthen the retrieval of contextual and event details.

The research presented here suggests that gaze reinstatement is not only a passive reflection of the contents of memory, but that it also actively facilitates further memory retrieval by reinstating the spatiotemporal encoding context. During encoding, eye movements serve to bind and encode spatial and temporal relations among objects and the context in which they are embedded. When that encoded representation is subsequently cued, gaze reinstatement facilitates the reactivation of further details by reinstating the spatiotemporal context that links them. Over the past several decades, models of overt visual attention have begun to incorporate top-down effects along with bottom-up effects to predict eye movements. However, the same cannot be said about models of memory. In fact, studies of memory retrieval rarely examine eye movements or the possible effects of eye movements on mnemonic processes and performance. Recently, however, some reviews have called for greater incorporation of eye tracking and eye movement analysis in memory research [104,105]. Extending these appeals, we suggest that future memory research not only control for measures such as the number and duration of fixations, but also consider gaze patterns and the similarity between them. As discussed previously, retrieval-related gaze reinstatement is significantly correlated with neural reinstatement [93], which is commonly used as a measure of memory. Thus, it is possible that reports of neural reinstatement may be partially explained by overlap in eye movements between encoding and retrieval. Understanding the relationship between gaze reinstatement and neural reinstatement and other mnemonic effects will be critical to advancing memory theories.

Evidence of gaze reinstatement in younger adults critically extends ideas regarding oculomotor control and memory. But, gaze reinstatement not only supports memory performance in younger adults. In fact, gaze reinstatement shows the largest memory effects in older adults, a population that typically shows declines in hippocampal integrity (e.g., volume, structural and functional connectivity) and related deficits in relational memory [73]. Yet, despite the potential for age-related memory improvement, research on gaze reinstatement in older adults is limited, with only a few studies investigating how functional gaze reinstatement changes across the adult lifespan. Given that age-related cognitive deficits are often accompanied by significant behavioral changes, identifying early markers of age-related cognitive decline and possible strategies for overcoming it are critical targets for cognitive neuroscience research. Gaze reinstatement has the potential to address both of these questions. Studies investigating gaze reinstatement in older adults have shown that older adults recruit gaze reinstatement to a greater extent than younger adults to support memory performance [36,37]. Gaze reinstatement may be particularly invoked by older adults to reinforce the spatiotemporal context, which older adults have difficulty establishing at encoding, and to reduce internal cognitive demands. This is consistent with a more general age-related shift from reliance on internal representations to reliance on the external world for environmental support [106]. Thus, given the memory-enhancing effects of gaze reinstatement, future research may help to determine whether healthy older adults, and older adults with dementia, can be taught, or otherwise biased towards gaze reinstatement in order to boost memory performance. Answering these questions will be critical for advancing theories of aging and memory, and for developing applied interventions for aging.

## 7. Conclusions

Just over fifty years ago, Hebb (1968) [25] and, shortly thereafter, Noton and Stark (1971) [27,28] suggested that gaze behavior is important not only for seeing the world around us, but also for imagining and remembering it. Decades later, technological and theoretical advances have now made clear that overt visual attention and memory are intimately related. Consistent with the predictions made by Hebb [25] and Noton and Stark [27,28], research has established that eye movements carry information, not only about visual attentional selection, but also about the contents of memory. Expanding on this link between visual exploration and memory, other work suggests that eye movements play an *active* role in memory encoding and retrieval, facilitating the mnemonic processes of relational binding and comparison by shifting attention within and between salient stimulus features, or the locations previously occupied by them. We propose that this gaze reinstatement is obligatorily recruited to support memory retrieval when task demands exceed mnemonic resources by actively reinstating the spatiotemporal context of encoded stimuli, which in turn facilitates access to and the retrieval of associated stimulus features via relational memory. Future work should continue to investigate the conditions under which gaze reinstatement supports memory, including task requirements (e.g., spatial versus temporal), mnemonic demands (e.g., short term memory maintenance versus long-term memory retrieval), goal states (e.g., recognition versus visualization), and individual differences in cognitive abilities (e.g., younger adults versus older adults). In addition, future research should explore the boundary limits of gaze reinstatement, or more specifically, how much or how little of the spatial or temporal context must be reinstated in order to facilitate memory retrieval under these different conditions. For example, tasks that are more spatial in nature, such as remembering the relative locations of items in a visual array, may rely more heavily on the reinstatement of spatial relations than temporal relations, whereas tasks that are more temporal in nature, such as remembering the relative order of the appearance of items in an array, may show the opposite effect. Moreover, we might expect that gaze would be more faithfully reinstated during image repetition, wherein the spatial index of the encoded stimulus is preserved, compared to visualization, whereas gaze reinstatement may play a more significant role in memory retrieval during visualization when lack of visual input increases reliance on internal cognitive processes. Ultimately, further research will be required to better understand potential nuances in the relationship between the quality and features of gaze reinstatement, and mnemonic performance, across different task conditions. Finally, combining eye movement monitoring with other methodologies including functional magnetic resonance imaging (fMRI), magnetoencephalography (MEG), and electroencephalography (EEG) will be critical to understanding the neural mechanisms underlying the beneficial effects of gaze reinstatement on memory. Finally, although many questions remain regarding the relationship between gaze reinstatement and memory retrieval, the discussed research serves as a foundation for advancing a comprehensive understanding of visual exploration and memory as fundamentally related processes.

## References

[1] Itti L., Koch C. (2000). A saliency-based search mechanism for overt and covert shifts of visual attention. Vis. Res..

[2] Yarbus A.L. (1967). Eye Movements and Vision.

[3] Castelhano M.S., Mack M.L., Henderson J.M. (2009). Viewing task influences eye movement control during active scene perception. J. Vis..

[4] Loftus G.R., Mackworth N.H. (1978). Cognitive determinants of fixat ion locati on during picture viewing. J. Exp. Psychol. Hum. Percept. Perform..

[5] Cerf M., Frady E.P., Koch C. (2009). Faces and text attract gaze independent of the task: Experimental data and computer model. J. Vis..

[6] Tatler B.W., Vincent B.T. (2009). The prominence of behavioural biases in eye guidance. Vis. Cogn..

[7] Einhäuser W., Perona P. (2008). Objects predict fixations better than early saliency. J. Vis..

[8] Torralba A., Oliva A., Castelhano M.S., Henderson J.M. (2006). Contextual guidance of eye movements and attention in real-world scenes: The role of global features on object search. Psy. Rev..

[9] Henderson J. (2003). Human gaze control during real-world scene perception. Trends Cogn. Sci..

[10] Henderson J.M., Hayes T.R. (2018). Meaning guides attention in real-world scene images: Evidence from eye movements and meaning maps. J. Vis..

[11] Chan J.P.K., Kamino D., Binns M.A., Ryan J.D. (2011). Can changes in eye movement scanning alter the age-related deficit in recognition memory?. Front. Psychol..

[12] Henderson J.M., Williams C.C., Falk R.J. (2005). Eye movements are functional during face learning. Mem. Cognit..

[13] Olsen R.K., Sebanayagam V., Lee Y., Moscovitch M., Grady C.L., Rosenbaum R.S., Ryan J.D. (2016). The relationship between eye movements and subsequent recognition: Evidence from individual differences and amnesia. Cortex.

[14] Olsen R.K., Lee Y., Kube J., Rosenbaum R.S., Grady C.L., Moscovitch M., Ryan J.D. (2015). The Role of Relational Binding in Item Memory: Evidence from Face Recognition in a Case of Developmental Amnesia. J. Neurosci..

[15] Liu Z., Shen K., Olsen R.K., Ryan J.D. (2017). Visual sampling predicts hippocampal activity. J. Neurosci..

[16] Killian N., Jutras M., Buffalo E. (2012). A Map of Visual Space in the Primate Entorhinal Cortex. Nature.

[17] Jutras M.J., Fries P., Buffalo E.A. (2013). Oscillatory activity in the monkey hippocampus during visual exploration and memory formation. Proc. Natl. Acad. Sci..

[18] Staudigl T., Hartl E., Noachtar S., Doeller C.F., Jensen O. (2017). Saccades are phase-locked to alpha oscillations in the occipital and medial temporal lobe during successful memory encoding. PLOS Biol..

[19] Althoff R., Cohen N. (1999). Eye-movement based memory effect: A reprocessing effect in face perception. J. Exp. Psychol. Learn. Mem. Cogn..

[20] Ryan J.D., Althoff R.R., Whitlow S., Cohen N.J. (2000). Amnesia is a Deficit in Relational Memory. Psychol. Sci..

[21] Hannula D.E., Ranganath C. (2009). The eyes have it: Hippocampal activity predicts expression of memory in eye movements. Neuron.

[22] Bridge D.J., Cohen N.J., Voss J.L. (2017). Distinct hippocampal versus frontoparietal-network contributions to retrieval and memory-guided exploration Donna. J. Cogn. Neurosci..

[23] Shen K., Bezgin G., Selvam R., McIntosh A.R., Ryan J.D. (2016). An Anatomical Interface between Memory and Oculomotor Systems. J. Cogn. Neurosci..

[24] Ryan J.D., Shen K., Kacollja A., Tian H., Griffiths J., McIntosh R. (2018). The functional reach of the hippocampal memory system to the oculomotor system. bioRxiv.

[25] Hebb D.O. (1968). Concerning imagery 1. Psychol. Rev..

[26] Sheehan P.W., Neisser U. (1969). Some variables affecting the vividness of imagery in recall. Br. J. Psychol..

[27] Noton D., Stark L. (1971). Scanpaths in saccadic eye movements while viewing and recognizing patterns. Vis. Res..

[28] Noton D., Stark L. (1971). Scanpaths in eye movements during pattern perception. Science.

[29] Blackmon T.T., Ho Y.F., Chernyak D.A., Azzariti M., Stark L.W., Rogowitz B.E., Pappas T.N. (1999). Dynamic scanpaths: Eye movement analysis methods. Human Vision and Electronic Imaging IV: SPIE proceedings.

[30] Choi Y.S., Mosley A.D., Stark L.W. (1995). String editing analysis of human visual search. Optom. Vis. Sci..

[31] Hacisalihzade S.S., Stark L.W., Allen J.S. (1992). Visual perception and sequences of eye movement fixations: A stochastic modeling approach. IEEE Trans. Syst. Man. Cybern..

[32] Brandt S., Stark L. (1997). Spontaneous eye movements during visual imagery reflect the content of the visual scene. J. Cogn. Neurosci..

[33] Foulsham T., Underwood G. (2008). What can saliency models predict about eye movements? Spatial and sequential aspects of fixations during encoding and recognition. J. Vis..

[34] Underwood G., Foulsham T., Humphrey K. (2009). Saliency and scan patterns in the inspection of real-world scenes: Eye movements during encoding and recognition. Vis. cogn..

[35] Holm L., Mäntylä T. (2007). Memory for scenes: Refixations reflect retrieval. Mem. Cogn..

[36] Wynn J.S., Olsen R.K., Binns M.A., Buchsbaum B.R., Ryan J.D. (2018). Fixation reinstatement supports visuospatial memory in older adults. J. Exp. Psychol. Hum. Percept. Perform..

[37] Wynn J.S., Bone M.B., Dragan M.C., Hoffman K.L., Buchsbaum B.R., Ryan J.D. (2016). Selective scanpath repetition during memory-guided visual search. Vis. Cogn..

[38] Ryals A.J., Wang J.X., Polnaszek K.L., Voss J.L. (2015). Hippocampal contribution to implicit configuration memory expressed via eye movements during scene exploration. Hippocampus.

[39] Damiano C., Walther D.B. (2019). Distinct roles of eye movements during memory encoding and retrieval. Cognition.

[40] Laeng B., Bloem I.M., D’Ascenzo S., Tommasi L. (2014). Scrutinizing visual images: The role of gaze in mental imagery and memory. Cognition.

[41] Laeng B., Teodorescu D.-S. (2002). Eye scanpaths during visual imagery reenact those of perception of the same visual scene. Cogn. Sci..

[42] Johansson R., Johansson M. (2013). Look here, eye movements play a functional role in memory retrieval. Psychol. Sci..

[43] Olsen R.K., Chiew M., Buchsbaum B.R., Ryan J.D. (2014). The relationship between delay period eye movements and visuospatial memory. J. Vis..

[44] Humphrey K., Underwood G. (2008). Fixation sequences in imagery and in recognition during the processing of pictures of real-world scenes. J. Eye Mov. Res..

[45] Bochynska A., Laeng B. (2015). Tracking down the path of memory: Eye scanpaths facilitate retrieval of visuospatial information. Cogn. Process..

[46] Spivey M.J., Geng J.J. (2001). Oculomotor mechanisms activated by imagery and memory: Eye movements to absent objects. Psychol. Res..

[47] Kumcu A., Thompson R.L. (2018). Less imageable words lead to more looks to blank locations during memory retrieval. Psychol. Res..

[48] Foulsham T., Kingstone A. (2013). Fixation-dependent memory for natural scenes: An experimental test of scanpath theory. J. Exp. Psychol. Gen..

[49] Reuter-lorenz P.A., Cappell K.A. (2008). Neurocognitive Aging and the Compensation Hypothesis. Curr. Dir. Psychol. Sci..

[50] Ferreira F., Apel J., Henderson J.M. (2008). Taking a new look at looking at nothing. Trends Cogn. Sci..

[51] Robin J. (2018). Spatial scaffold effects in event memory and imagination. Wiley Interdiscip. Rev. Cogn. Sci..

[52] Rondina R., Curtiss K., Meltzer J.A., Barense M.D., Ryan J.D. (2017). The organisation of spatial and temporal relations in memory. Memory.

[53] Tulving E., Thomson D.M. (1973). Encoding specificity and retrieval processes in episodic memory. Psychol. Rev..

[54] Kent C., Lamberts K. (2008). The encoding-retrieval relationship: Retrieval as mental simulation. Trends Cogn. Sci..

[55] Parker R.E. (1978). Picture processing during recognition. J. Exp. Psychol. Hum. Percept. Perform..

[56] Richardson D.C., Spivey M.J. (2000). Representation, space and Hollywood Squares: Looking at things that aren’t there anymore. Cognition.

[57] Altmann G.T.M. (2004). Language-mediated eye movements in the absence of a visual world: The “blank screen paradigm”. Cognition.

[58] Johansson R., Holsanova J., Holmqvist K. (2006). Pictures and spoken descriptions elicit similar eye movements during mental imagery, both in light and in complete darkness. Cogn. Sci..

[59] Johansson R., Holsanova J., Dewhurst R., Holmqvist K. (2012). Eye movements during scene recollection have a functional role, but they are not reinstatements of those produced at encoding. J. Exp. Psychol. Hum. Percept. Perform..

[60] Scholz A., Mehlhorn K., Krems J.F. (2016). Listen up, eye movements play a role in verbal memory retrieval. Psychol. Res..

[61] Martarelli C.S., Mast F.W. (2013). Eye movements during long-term pictorial recall. Psychol. Res..

[62] Parkhurst D., Law K., Niebur E. (2002). Modeling the role of salience in the allocation of overt visual attention. Vis. Res..

[63] Tatler B.W., Baddeley R.J., Gilchrist I.D. (2005). Visual correlates of fixation selection: Effects of scale and time. Vis. Res..

[64] O’Connell T.P., Walther D.B. (2015). Dissociation of salience-driven and content-driven spatial attention to scene category with predictive decoding of gaze patterns. J. Vis..

[65] Castelhano M.S., Henderson J.M. (2007). Initial scene representations facilitate eye movement guidance in visual search. J. Exp. Psychol. Hum. Percept. Perform..

[66] Chun M.M., Jiang Y. (1998). Contextual cueing: Implicit learning and memory of visual context guides spatial attention. Cogn. Psychol..

[67] Chau V.L., Murphy E.F., Rosenbaum R.S., Ryan J.D., Hoffman K.L. (2011). A Flicker Change Detection Task Reveals Object-in-Scene Memory Across Species. Front. Behav. Neurosci..

[68] Myers C.W., Gray W.D. (2010). Visual scan adaptation during repeated visual search. J. Vis..

[69] Shih S.-I., Meadmore K.L., Liversedge S.P. (2012). Aging, eye movements, and object-location memory. PLoS ONE.

[70] Brandstatt K.L., Voss J.L. (2014). Age-related impairments in active learning and strategic visual exploration. Front. Aging Neurosci..

[71] Vieweg P., Riemer M., Berron D., Wolbers T. (2018). Memory Image Completion: Establishing a task to behaviorally assess pattern completion in humans. Hippocampus.

[72] Old S.R., Naveh-Benjamin M. (2008). Differential effects of age on item and associative measures of memory: A meta-analysis. Psychol. Aging.

[73] Grady C.L., Ryan J.D., Hannula D.E., Duff M.C. (2017). Age-Related Differences in the Human Hippocampus: Behavioral, Structural and Functional Measures. The Hippocampus from Cells to Systems.

[74] Grady C. (2012). The cognitive neuroscience of ageing. Nat. Rev. Neurosci..

[75] Stern Y. (2009). Cognitive reserve. Neuropsychologia.

[76] Cohen N.J., Eichenbaum H. (1993). Memory, Amnesia, and the Hippocampal System.

[77] Eichenbaum H., Cohen N.J. (2001). From Conditioning to Conscious Recollection: Memory Systems of the Brain.

[78] Moses S.N., Ryan J.D. (2006). A comparison and evaluation of the predictions of relational and conjunctive accounts of hippocampal function. Hippocampus.

[79] Eichenbaum H., Otto T., Cohen N.J. (2010). Two functional components of the hippocampal memory system. Behav. Brain Sci..

[80] Ryan J.D., Cohen N.J. (2004). The nature of change detection and online representations of scenes. J. Exp. Psychol. Hum. Percept. Perform..

[81] Ryan J.D., Cohen N.J. (2003). Evaluating the neuropsychological dissociation evidence for multiple memory systems. Cogn. Affect. Behav. Neurosci..

[82] Olsen R.K., Moses S.N., Riggs L., Ryan J.D. (2011). The hippocampus supports multiple cognitive processes through relational binding and comparison. Front. Hum. Neurosci..

[83] Yassa M.A., Stark C.E.L. (2011). Pattern separation in the hippocampus. Trends Neurosci..

[84] Hunsaker M.R., Kesner R.P. (2013). The operation of pattern separation and pattern completion processes associated with different attributes or domains of memory. Neurosci. Biobehav. Rev..

[85] Liu K.Y., Gould R.L., Coulson M.C., Ward E.V., Howard R.J. (2016). Tests of pattern separation and pattern completion in humans—A systematic review. Hippocampus.

[86] Hannula D.E., Ryan J.D., Warren D.E. (2017). Beyond Long-Term Declarative Memory: Evaluating Hippocampal Contributions to Unconscious Memory Expression, Perception, and Short-Term Retention. The Hippocampus from Cells to Systems.

[87] Rolls E.T., Wirth S. (2018). Spatial representations in the primate hippocampus, and their functions in memory and navigation. Prog. Neurobiol..

[88] Eichenbaum H. (2014). Time cells in the hippocampus: A new dimension for mapping memories. Nat. Rev. Neurosci..

[89] Cohen N.J., Ryan J., Hunt C., Romine L., Wszalek T., Nash C. (1999). Hippocampal system and declarative (relational) memory: Summarizing the data from functional neuroimaging studies. Hippocampus.

[90] Staresina B.P., Davachi L. (2009). Mind the Gap: Binding Experiences across Space and Time in the Human Hippocampus. Neuron.

[91] Henke K., Buck A., Weber B., Wieser H.G. (1997). Human Hippocampus Establishes Associations in Memory. Hippocampus.

[92] Mayes A., Montaldi D., Migo E. (2007). Associative memory and the medial temporal lobes. Trends Cogn. Sci..

[93] Bone M.B., St-Laurent M., Dang C., McQuiggan D.A., Ryan J.D., Buchsbaum B.R. (2018). Eye Movement Reinstatement and Neural Reactivation During Mental Imagery. Cereb. Cortex.

[94] Wolfe J.M. (1994). Guided Search 2.0 A revised model of visual search. Psychon. Bull. Rev..

[95] Hamker F.H. (2006). Modeling feature-based attention as an active top-down inference process. Biosystems.

[96] Klein R.M. (2000). Inhibition of return. Trends Cogn. Sci..

[97] Thompson K.G., Biscoe K.L., Sato T.R. (2005). Neuronal Basis of Covert Spatial Attention in the Frontal Eye Field. J. Neurophysiol..

[98] Ipata A.E., Gee A.L., Goldberg M.E., Bisley J.W. (2006). Activity in the Lateral Intraparietal Area Predicts the Goal and Latency of Saccades in a Free-Viewing Visual Search Task. J. Neurosci..

[99] Shen K., Paré M. (2007). Neuronal activity in superior colliculus signals both stimulus identity and saccade goals during visual conjunction search. J. Vis..

[100] Fecteau J.H., Munoz D.P. (2006). Salience, relevance, and firing: A priority map for target selection. Trends Cogn. Sci..

[101] Bisley J.W., Mirpour K. (2019). The neural instantiation of a priority map. Curr. Opin. Psychol..

[102] Shen K., McIntosh A.R., Ryan J.D. (2014). A working memory account of refixations in visual search. J. Vis..

[103] Meister M.L.R., Buffalo E.A. (2016). Getting directions from the hippocampus: The neural connection between looking and memory. Neurobiol. Learn. Mem..

[104] Hannula D.E., Althoff R.R., Warren D.E., Riggs L., Cohen N.J. (2010). Worth a glance: Using eye movements to investigate the cognitive neuroscience of memory. Front. Hum. Neurosci..

[105] Voss J.L., Bridge D.J., Cohen N.J., Walker J.A. (2017). A Closer Look at the Hippocampus and Memory. Trends Cogn. Sci..

[106] Lindenberger U., Mayr U. (2014). Cognitive aging: Is there a dark side to environmental support?. Trends Cogn. Sci..

